# Pharmaceuticals and Personal Care Products across Different Water Bodies in Taihu Lake Basin, China: Occurrence, Source, and Flux

**DOI:** 10.3390/ijerph191711135

**Published:** 2022-09-05

**Authors:** Jichao Huang, Jiannan Ding, Hang Jiang, Zhenguo Wang, Lixing Zheng, Xiaojun Song, Hua Zou

**Affiliations:** 1 School of Environmental and Civil Engineering, Jiangnan University, Wuxi 214122, China; 2 Jiangsu Collaborative Innovation Center of Technology and Material of Water Treatment, Suzhou 215009, China; 3 Biomass Energy and Biological Carbon Reduction Engineering Center of Jiangsu Province, Wuxi 214122, China

**Keywords:** PPCPs, TLB, occurrence, source apportionment, flux

## Abstract

Although pharmaceuticals and personal care products (PPCPs) have attracted great attentions, their occurrence characteristics across different water bodies at a basin scale remain poorly understood. To grasp a more comprehensive understanding of PPCP pollution from the perspective of the whole basin, the occurrence, spatial and seasonal variation, source, and flux of thirteen PPCPs across the different environmental compartments of the northern Taihu Lake Basin (TLB) were studied. The results showed that the non-therapeutic pharmaceuticals caffeine (CFI) and n, n-diethyl-m-toluamide (DEET) were the main components across the different environmental compartments. The total concentrations of detected PPCPs ranged from 0.2 to 2437.9 ng/L. Higher concentrations of PPCPs were observed in spring and autumn, which were mainly attributed to seasonal differences in PPCP consumption. Generally, pollution level was higher in industry and agriculture area and in the inner bay and southwest of Taihu Lake. Source apportionment indicated that untreated water was the main source of PPCPs in river waters of the northern TLB. Flux estimation showed that the mean annual flux of PPCPs from northern TLB to Taihu Lake in 2021 was 1.6 t/a, which was higher in comparison with other areas. Overall, the resulting data will be useful to enrich the research of PPCPs in freshwater for environmental investigations.

## 1. Introduction

Pharmaceuticals and personal care products (PPCPs) are constantly being released into aquatic environments via multiple pathways, including domestic wastewater, hospital discharges, improper manufacturer disposal, agricultural runoff, and wastewater treatment plants (WWTPs) [1]. Although PPCPs are present at concentrations of ng/L–μg/L in natural water bodies [2,3], numerous studies have suggested that PPCPs can cause adverse effects on non-target aquatic organisms [4]. Hence, PPCPs have been of increasing environmental concern in recent decades.

PPCPs are generally low-volatile, highly polar, and hydrophilic in nature [5]. They may migrate across different environmental compartments via aqueous transport and food chain dispersal [6]. The presence of PPCPs across different environmental compartments has been widely investigated in recent years; however, most studies have focused on specific environmental compartments, such as rivers [7,8], lakes [9,10], and WWTPs [11,12]. Only a few studies have comprehensively investigated the occurrence of PPCPs across different environmental compartments at a basin scale. A few studies have revealed that across different basins, the hotspots of PPCPs could vary owing to different socioeconomic and hydrological conditions. For example, Wu et al. [13] found that in the Yangtze River Basin, the mean concentration of caffeine (CFI) in lakes (210.7 ± 301.2 ng/L) was generally higher than that in rivers (142.0 ± 174.3 ng/L). In contrast, the mean concentration of CFI in rivers (349.2 ± 173.5 ng/L) was higher than that in lakes (85.0 ± 28.6 ng/L) in the Qingshan Lake Basin [14]. In addition, differences in the occurrence of PPCPs may be attributed to seasonal variations. Considerable seasonal variations in PPCPs have been revealed in WWTP influents and effluents [11,15], rivers [16,17], and lakes [18,19]. Therefore, seasonal variations involving different environmental compartments should be integrated into account to clarify the occurrence characteristics of PPCPs in an entire basin.

Taihu Lake is the third-largest freshwater lake in China. The Taihu Lake Basin (TLB) is located in the Yangtze River Delta and covers an area of approximately 3.69 × 10^4^ km^2^. The basin is one of the most developed areas in China, with a population of 67.55 million and a total gross domestic product (GDP) of RMB 9997.8 billion in 2020 [20]. It is a highly urbanized and ecologically sensitive zone [21]. Along with the rapid economic development and intensive use of water resources, the water quality of the TLB has deteriorated. It has been concluded that 36 PPCPs were detected in Taihu Lake and the concentrations were up to hundreds of ng/L [22]. In 12 typical inflow rivers of Taihu Lake, seven out of ten typical PPCPs were detected, with concentrations as high as 94.91 ng/L [23]. Moreover, 32 targeted PPCPs were detected in influents and effluents of 9 WWTPs around Taihu Lake, with total concentrations ranging from 204.3 to 35,321.9 ng/L [24]. These results suggest that PPCPs ubiquitously exist in the various environmental compartments of the TLB. However, the occurrence characteristic of PPCPs in the basin scale remains unclear.

As mentioned above, understanding how patterns of PPCPs vary over space and time is an important step to better evaluating the PPCP pollution at the basin scale. Moreover, an understanding of the sources and fluxes of PPCPs from land waters to the lake area is critical to inform mitigation strategies for the PPCP pollution in the TLB. Several studies have indicated that wastewater effluent is a major source of PPCPs within a basin [25,26]. Meanwhile, other emission sources, such as untreated wastewater, could also make large contributions to the total load of PPCPs in rivers and lakes [27,28]. Hence, PPCPs can be released from different sources (e.g., agricultural runoff, untreated water, and WWTP effluent) within a given basin, and the main emission sources of PPCPs in the waters in the TLB are still an inconclusive issue [29]. More efforts should be made to clarify the emission sources of PPCPs in the TLB. With respect to the PPCP flux, information on it within the TLB is also limited. Previous studies have suggested that PPCP flux could vary from a few to hundreds kg/a across different basins, such as in the cases of the Ugie River Basin (4.5–4.6 kg/a) [30] and Shijing River Basin (194.1 kg/a) [31]. Recently, Jiang et al. [8] estimated that 21.9–211.7 kg of PPCPs were annually released into Taihu Lake from the Taige Canal. An et al. [24] found that PPCP flux from the Wujin District to Taihu Lake was 168.9 kg/a. However, the existing research arranged only one or two inflow rivers in the northwest part of the TLB, therefore, more inflow rivers should be investigated to represent the pollution load of PPCPs in the entire northern TLB.

To address the above issues, this study takes Taihu Lake and various land waters in the northern TLB as the research area and aims to (1) comprehensively evaluate the spatial and seasonal variations of PPCP occurrence across different environmental compartments of the TLB and (2) clarify the source and flux of PPCPs in the basin. To this end, a series of monitoring campaigns was conducted from March 2019 to January 2022. Water samples were collected from various environmental compartments in the northern TLB, including influents and effluents of WWTPs, rivers, and lakes. The occurrences of 13 target PPCPs, namely roxithromycin (ROX), clarithromycin (CLR), fluoxetine (FLX), citalopram (CTP), sertraline (SER), metoprolol (MTL), bezafibrate (BZB), gemfibrozil (GFB), n, n-diethyl-m-toluamide (DEET), triclocarban (TCC), clotrimazole (CTM), CFI, and carbamazepine (CBZ) were investigated. This study can provide a comprehensive picture of the occurrence of PPCPs in the aquatic environment of the TLB.

## 2. Materials and Methods

### 2.1. Chemicals and Reagents

The 13 target PPCPs belong to different categories, including antibiotics, antidepressants, antihypertensive, lipid regulators, insect repellent, fungicides, antimicrobial agents, stimulants, and antiepileptic agents. They were selected in this study due to their common use in the TLB. Moreover, the selected compounds have been widely discovered and are considered as high risk in surface waters in China [8,25,28]. Standards of 13 PPCPs with high purity grade (≥99%) were purchased from Dr. Ehrenstorfer (Augsburg, Germany). The physical-chemical properties of the target compounds are listed in Table S1 (Supplementary Material). Stock solutions were prepared in methanol and stored at −20 °C in the dark. Working solutions were obtained by serial dilution prior to use. The methanol and acetone used in the chemical analysis procedures were HPLC grade and obtained from Fisher (Fair Lawn, NJ, USA).

### 2.2. Sample Collection and Pretreatment

In our monitoring campaigns, various water samples, including influents and effluents of WWTPs, rivers, and lakes were collected by grab sampling. Therein, two WWTPs (denoted as WWTP A and B) in northern TLB were selected and four sampling events were performed for their influent and effluent (WWTP A: August 2019 and December 2019; WWTP B: July 2021 and January 2022). In WWTP A, a Orbal oxidation ditch (OD) process and a membrane bioreactor (MBR) process are employed in parallel. The two processes are fed with the same raw wastewater. In WWTP B, an anaerobic-anoxic-aerobic (A^2^O) process is employed. All the three processes (i.e., OD, MBR, and A^2^O) were operated efficiently during our sampling. The schematic diagram of the three treatment processes in WWTP A and B is shown in Figure S1. In each sampling event for WWTP A, one sample of the influent was collected from the raw wastewater and two samples of the effluents were respectively collected from the OD and MBR processes. For the WWTP B, two samples were respectively collected from the influent and effluent of the A^2^O process. In the four sampling events for the two WWTPs, a total of 10 samples were collected.

**Figure 1:**
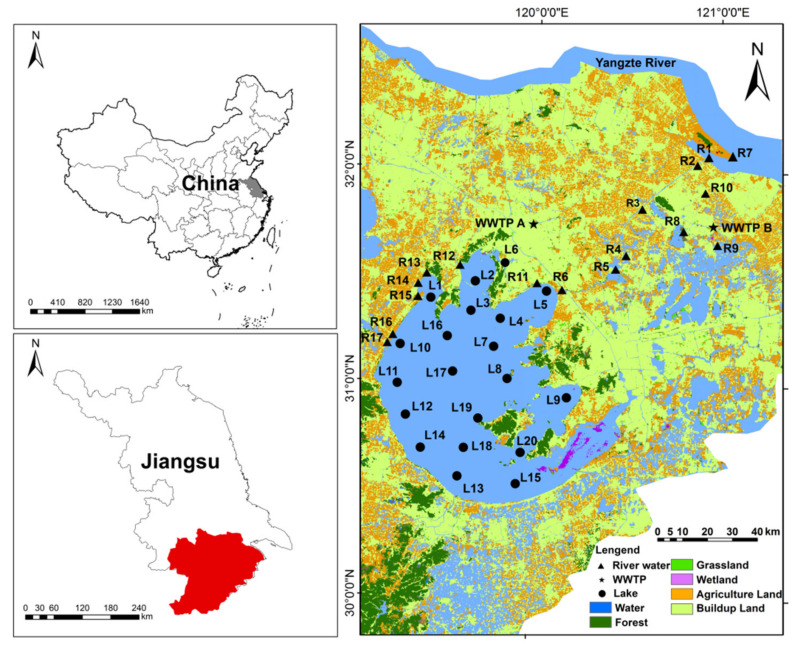
The land use and the location of sampling sites in the TLB.

In the present study, four sampling events for rivers (April 2021, July 2021, October 2021, and January 2022) (in total n = 61) (Samples in R11–R17 were not collected in April 2021 due to COVID-19 pandemic) and four sampling events for Taihu Lake (March 2019, June 2019, September 2019, and December 2019) (in total n = 78) (Samples in L1 were not collected in June and September 2019 due to maintenance of the ship) were conducted. Geographical coordinates of each sampling site (Figure 1) were recorded with a handheld global positioning system (GPS) and the detailed geographical information of the sampling sites are listed in Table S2.

The water sample was collected in 1 L amber glass bottles by a water collector. Therein, the samples in the rivers and lakes were from 50 cm below the water surface. To avoid potential biodegradation, 1% of methanol was added into the samples. The amber glass bottles were pre-rinsed with the surface water for three times before sample collection. After collection, the water samples were transferred on ice to laboratory within 3 h.

### 2.3. Analysis of PPCPs

In laboratory, all collected water samples were first filtered through glass fiber filters (Whatman, GF/C, 0.45 μm, Bedford, MA, USA) to remove suspended particles. The samples were then extracted using Waters Oasis HLB cartridges (6 mL, 200 mg, Waters, Milford, MA, USA) and the related parameters are shown in Table S3. The eluents were concentrated to near dryness under a gentle stream of nitrogen. The resulting residues were re-dissolved in 1 mL of methanol aqueous solution. After filtration through a 0.22 μm membrane to remove particles, the final extracts were transferred to 2-mL amber glass vials in preparation for analysis.

The PPCPs were analyzed by a Waters Acquity UPLC-MS/MS system with an electrospray ionization source (ESI) in both positive and negative ionization modes. An ACQUITY UPLC BEH-C18 column (100 mm × 1.7 μm × 2.1 mm; Waters, Milford, MA, USA) was employed and maintained at 40 °C. An injection volume of 5 μL was used for each analysis, and the flow rate was 0.3 mL/min. Detailed parameters of the chromatographic and mass spectrometry conditions for the target compounds are presented in Tables S4 and S5, respectively. The limit of detection (LOD) and the limit of quantification (LOQ) for each analyte were defined as the concentrations corresponding to signal-to-noise (S/N) ratios of 3 and 10, respectively. The LOD and LOQ were reported in the ranges of 0.03–3.68 ng/L and 0.1–11.21 ng/L, respectively. The concentration was set as ND when it was lower than LOD; when the concentration was between LOD and LOQ, it was set as half of LOQ.

### 2.4. Quality Control

The samples were quantitatively analyzed by external standard method, and mixed solutions were prepared by various target standard compounds. A six-point standard calibration curve was prepared for each analyte at concentrations from 1 to 1000 ng/L. The correlation coefficients (r^2^) of the calibration curves were >0.99 for most analytes. Three ultrapure water samples spiked with all target compounds were analyzed using the same procedure as that for real water samples to validate recovery. The recoveries for analytes were in the range of 66.6–112.8%.

### 2.5. Statistical Analysis

Numerical data are presented as the mean ± standard deviation (SD). Data analysis was performed by SPSS 26 (SPSS Company, Chicago, IL, USA). A one-way ANOVA was performed to determine whether the differences in PPCP concentrations between groups were significant. When significant differences were detected (at α = 0.05), a post hoc Duncan test was carried out to perform pairwise comparisons between group means, since the sample sizes of the groups were unequal. Before one-way ANOVA, the Shapiro–Wilk normality test and Levene test for homogeneity of variance were carried out using untransformed or transformed data. For data with inhomogeneous variance or an abnormal distribution (*p* ≤ 0.05), the Mann–Whitney U test (two groups) or Kruskal–Wallis test (more than two groups) was performed instead. The differences were regarded as significant at * *p* < 0.05 and extremely significant at ** *p* < 0.01 in all cases.

Source apportionment analysis was conducted using principal component analysis followed by multiple linear regression (PCA-MLR) with SPSS 26 (SPSS Company, Chicago, IL, USA). In the factor analysis model, PCA was used as the extraction method with Kaiser Normalization and varimax rotation. Only factors with eigen values >1 were used for identification of the possible sources. The relationship between the principal component and the chemical compound is indicated by the factor loadings. Stepwise MLR was then performed on the significant factors to determine the mass apportionment of each source to total concentration. After normalization, the basic equation of a multiple linear model is

(1) $${\overset{\wedge }{Z}}_{\mathrm{SumPPCP}}=\sum B_{k}t_{k}$$

where SumPPCP is the total concentrations of target PPCPs in this study, $\hat{Z}$ is the standard normalized deviate of the SumPPCP values, *B_k_* is the modeled regression coefficient, and *t_k_* is the factor score calculated by PCA.

The mean percentage contributions of each factor are calculated by Equation (2).

Mean contribution of source

(2) $$k(\%)=100\times \left(B_{k}/{\sum B}_{k}\right)$$

The eight typical inflow rivers are Wangyu, Li, Wujingang, Taige, Yincungang, Hengtang, Shedugang, and Dapu. Water flow from northern TLB to Taihu Lake were acquired from the government hydrologic website (http://www.tba.gov.cn/slbthlyglj/sj/sj.html, accessed on 4 July 2022). The annual flux of PPCPs into the lake is calculated as follows:

(3) $$F_{I}=C_{ij}\times Q_{j}\times 10^{-9}$$

where *F_I_*(kg/a) is the annual flux of PPCPs from the inflow rivers in the northern TLB to Taihu Lake in 2021, *C_ij_* (ng/L) is the concentration of PPCPs in sampling sites, and *Q_j_* (m^3^) is the water flow from northern TLB to Taihu Lake in 2021.

## 3. Results and Discussion

### 3.1. Occurrence of PPCPs in the Northern TLB and the Lake Area

A total of four influent samples were collected during the WWTP samplings. Among the 13 target PPCPs, 6 and 9 PPCPs were detected in the influents of WWTP A and B, respectively (Table 1). The concentrations of all the detected PPCPs during the sampling period were in the range of 878.0–2437.9 and 119.3–491.6 ng/L for WWTP A and B, respectively. The target PPCPs in the influents were dominated by CFI, with a relative abundance of 69.5% for WWTP A and 72.5% for WWTP B. DEET was another compound that exhibited high concentrations with the percentage values of 18.5% and 19.9% for WWTP A and WWTP B, respectively. Despite the similar composition, the concentrations of most of the PPCPs, such as CFI, were significantly higher in the influents of WWTP A than those found in WWTP B (*p* < 0.05). The difference could be attributed to the varying types of wastewater entering WWTPs. The WWTP A is located in the urban areas of the city and the main inflow is the residential wastewater with the percentage of 70%. The WWTP B is located in the suburb areas of the city and industrial wastewaters account for 50% of the inflows. Moreover, as an indicator of human activities [32], the significantly greater levels of CFI observed at WWTP A further demonstrated that the higher proportion of residential wastewater treated in WWTP A than that treated in WWTP B.

**Figure 2:**
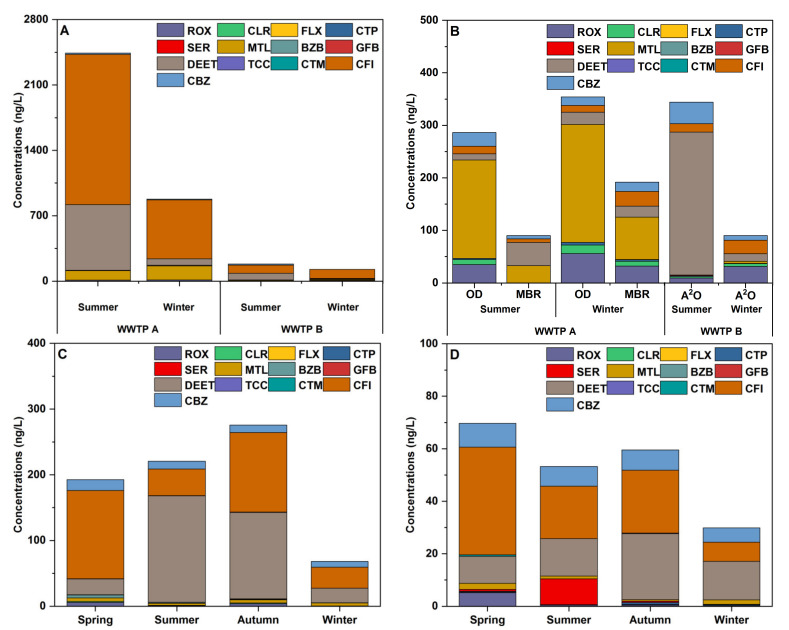
Seasonal variations in the concentrations of PPCPs in the wastewater (**A**) influents and (**B**) effluents, (**C**) river networks, and (**D**) Taihu Lake.

A total of six effluent samples were collected during the sampling period. Among the 13 target PPCPs, 8 and 10 PPCPs were detected in the effluents of WWTP A and B, respectively (Table 1). More kinds of PPCPs were found in the effluents of the two WWTPs than those in the influents. In this regard, the hydraulic lag in the treatment process was not considered for our sample collection, and thus, detecting different substances in the influent than in the effluent leads to the assumption that there might be more kinds of PPCPs in the raw waters during the previous days. All the target PPCPs detected during the sampling period were in the range of 90.4–354.2 and 61.3–344.2 ng/L for WWTP A and B, respectively (Figure 2B). For WWTP A, MTL, DEET, and ROX were the dominant compounds in the effluent, with percentages of 51.7%, 17.7%, and 11.2%, respectively. For WWTP B, DEET, CFI, and CBZ were the dominant compounds in the effluent, with percentages of 42.5%, 16.7%, and 15.9%, respectively. Although the concentrations of the PPCPs in the effluents were relatively low when compared to the samples in the influents, the daily treatment capacities of the two WWTPs were 100,000 and 40,000 tons, respectively. Thus, the daily PPCP load from the effluents of the two WWTPs to the receiving water was estimated as 11.5–367.8 g/d.

The removal rates of the PPCPs in the WWTPs can be calculated based on the concentrations of influents and effluents (Table S6). In the present study, the calculations of all the removal rates were based on grab samples that were not sampled with a hydraulic lag. Therefore, some discrepancy might be brought in due to diurnal variation of the concentration. In further studies, 24-h composite samples that are lagged by hydraulic retention time (HRT) should be collected and analyzed to better illustrate removal rates in the WWTPs [33]. Furthermore, the low detection frequency of some PPCPs made it difficult to generate statistically good results for individual WWTPs; thus, they were excluded from this section. In this study, CFI can be efficiently removed by both of the two WWTPs with the removal rates over 70% (Table S6). Consistently high removal efficiencies of CFI have been observed in the WWTPs in other countries (e.g., New Zealand [34] and Greece [35]). From the perspective of the WWTP processes, the removal efficiencies of all PPCPs were 78.2–96.3% by MBR, 59.7–88.3% by OD process, and −86.4–28.7% by A^2^O process. This indicates that the MBR process has a better treatment effect on the PPCPs. In addition, the transformation of the conjugated forms into the free form by microorganisms, as well as the grab sampling strategy adopted in this study, might explain the apparent negative removal efficiencies [11,36].

A total of 61 water samples were collected in the rivers of northern TLB during the sampling period. Among the 13 target PPCPs, 11 PPCPs were identified and quantified in the river water (Table 2). The concentrations of the PPCPs ranged between 0.7 and 971.2 ng/L. The compounds most frequently detected were DEET and CFI, with a detection frequency of 100% followed by CBZ (97.1%), ROX (95.6%), MTL (94.6%), CLR (70.6%), BZB (69.1%), and CTM (52.4%). FLX and TCC were not detected in any river water samples. Among the mean concentrations of all the PPCPs, DEET had the highest mean concentration (92.0 ± 145.7 ng/L), followed by CFI (76.0 ± 123.5 ng/L). The mean concentrations of the other PPCPs ranged ND–4.5 ng/L, which were quite lower than those of DEET and CFI. This fraction pattern of PPCP individuals in the surface water was significantly different from the WWTP effluents, but similar to that in the WWTP influents, indicating that the occurrences of PPCPs in the river networks in the TLB may significantly be contributed by untreated water. In recent years, CFI and DEET have been found to be the predominant PPCP contaminants in various river waters around the world, such as the rivers in New Zealand [37], Spain [38], and China [24,39]. CFI is widely found in coffee, tea, and other drinks. DEET is primarily used as an insect repellent for humans and this is the main source of this compound in aquatic environments [40]. With a population of more than 20 million people around the TLB [22], there could be significant consumptions of CFI and DEET in the study area.

A total of 78 water samples were collected in Taihu Lake during the sampling period. Among the 13 target PPCPs, 9 PPCPs were identified and quantified in the water samples of Taihu Lake (Table 3). The concentrations of the PPCPs ranged between 0.2 and 169.4 ng/L in different samples. The compounds most frequently detected in Taihu Lake were DEET, with a detection frequency of 100%, followed by CFI (98.7%), CBZ (89.7%), ROX (79.5%), MTL (65.4%), and SER (56.4%). No FLX, BZB, GFB, or TCC were detected in Taihu Lake. Among the mean concentrations of all the PPCPs, CFI had the highest mean concentration (22.7 ± 29.5 ng/L), followed by DEET (15.9 ± 12.3 ng/L). The mean concentrations of the other PPCPs ranged ND–7.6 ng/L. These results were consistent with most PPCP monitoring results in other lakes (e.g., Dongting Lake [9], Michigan Lake [41], and Vanern Lake [42]), where DEET and CFI were the predominant non-antibiotic compounds. In this study, lower environmental loads of PPCPs were observed in Taihu Lake than those in WWTPs (61.3–2440.7 ng/L) and rivers (23.2–1044.9 ng/L). However, the composition profile of PPCPs in Taihu Lake was similar to that in the river water, indicating that river networks could be an important source of PPCPs entering into the lake.

### 3.2. Seasonal Variation of PPCPs in the Northern TLB and the Lake Area

The total PPCP concentrations in summer (August 2019 for WWTP A and July 2021 for WWTP B) were significantly higher than that in winter (December 2019 for WWTP A and January 2022 for WWTP B) in the influent of WWTPs (*p* < 0.05) (Figure 2A). It is likely because of the high consumption of DEET and CFI in the summer [5,8]. In addition, MTL concentrations were higher in winter, with the mean concentrations 1.2–1.5 times greater than those in summer. The high concentration of MTL in winter was consistent with the fact that blood pressure has been shown to be higher in winter [43], and thus, an increased dosage of antihypertensive agents is expected. No significant differences by season were observed for other PPCPs in the WWTP influents.

The total PPCP concentration was significantly higher in summer than in winter in the WWTP B effluent (*p* < 0.05), which was consistent with the seasonal variation of the influent. In the WWTP A effluent, however, it was significantly higher in winter than in summer (*p* < 0.05) (Figure 2B). The PPCP concentrations in the WWTP effluent could be affected by several factors. In WWTP A, OD process is applied as one of the treatment processes, and the concentrations of PPCPs in the effluents of OD process were higher than that in the effluents of MBR process. The mean temperature in the TLB is generally under 5 °C in winter, which is unfavorable for activated sludge. This is likely an important factor for the inefficient removal and in consequence the high PPCP concentrations in the effluents in winter [44].

The seasonal variations of 13 target PPCPs in river waters of northern TLB are shown in Figure 2C. The highest total concentration of PPCPs was observed in autumn (October 2021), followed by summer (July 2021), spring (April 2021), and winter (January 2022). This seasonal variation should be caused by some specific PPCPs. Individually, DEET showed much higher concentrations in summer (161.9 ng/L) and autumn (131.6 ng/L) than those in spring (24.0 ng/L) and winter (22.3 ng/L). Similarly, Jiang et al. [8] observed that the mean concentration of DEET in summer was more than twice than that in winter in the river water of Taige Canal basin, which is located in the southern China. In the northern China, Meng et al. [45] observed that DEET had the highest concentration (2643.0 ng/L) in summer in the Beiyun River. The high concentrations of DEET were also reported in surface waters from Indonesia (up to 24,000 ng/L) in Southeast Asia [46]. High concentrations of DEET in the summer might be due to the increased usage of repellents [47], primarily the gray water from bathing or washing clothes following the application of DEET by humans [48]. CFI showed higher concentrations in spring (134.3 ng/L) and autumn (121.0 ng/L) than in summer (40.6 ng/L) and winter (32.0 ng/L). Changes in human social activities during different seasons likely affect the anthropogenic inputs of CFI in the environment [49]. An increase in the number of population owing to seasonal tourism could significantly contribute to the high concentration of CFI during spring and autumn. In addition, slightly high concentrations of ROX and CLR were observed during winter than during summer, although the differences were not significant (*p* > 0.05). ROX and CLR are primarily used to treat respiratory tract infections, and the frequent detection of these PPCPs can be explained by their universality in colder months. The concentrations of other PPCPs did not significantly change from season to season.

The seasonal variations of 13 target PPCPs in Taihu Lake are shown in Figure 2D. In Taihu Lake, the lowest total concentration of PPCPs was observed in winter. In the TLB, the precipitation in winter is generally less than that in other seasons, which can contribute to a low water level. Unlike the present study, several studies have suggested that the low water level in winter could concentrate PPCPs and thus elevate the pollution level in aquatic environments [50,51]. Here, it is conceivable to believe that the weak riverine input is the significant causative factor for the low concentration of PPCPs in winter in Taihu Lake. Our results suggest that in the TLB, the seasonal variation of PPCPs in Taihu Lake is governed by the riverine input, highlighting the importance of understanding the occurrences of PPCPs across different water bodies at a basin scale.

### 3.3. Spatial Distribution of PPCPs in the Northern TLB and the Lake Area

#### 3.3.1. Spatial Distribution

**Figure 3:**
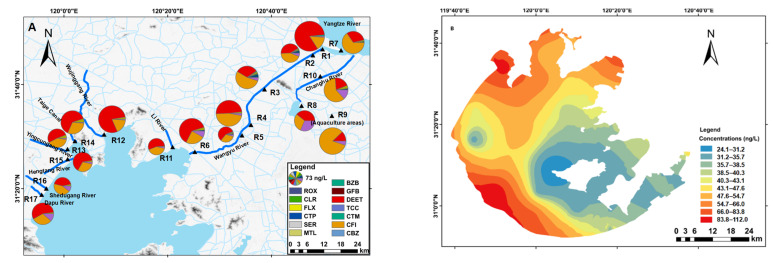
Spatial distributions in the concentrations of PPCPs in the (**A**) river networks and (**B**) Taihu Lake.

Concentrations of PPCPs at different sites in the river water are shown in Figure 3A. The highest total concentration of PPCPs was observed at R1 (335.5 ± 480.1 ng/L), followed by R12 (274.2 ± 252.0 ng/L), R9 (265.3 ± 397.9 ng/L), and R4 (251.2 ± 194.9 ng/L). R1 is located in a semienclosed area in the downstream of Yangtze River. The capacities of PPCP diffusion and self-purification of water body in this area could be limited, as a result of the poor hydrodynamic exchange. Therefore, the PPCP concentration at this site could constantly increase by long-term accumulation of the contaminants from the upstream of the Yangtze River. R12 is located in Wujin District, which is listed as one of the typical small and medium-sized cities in China for its developed industry and agriculture systems [28]. A large amount of PPCPs would be consumed in this district. Water samples from R9 were collected from the aquaculture pond located in Shajiabang. Shajiabang is a famous tourist location with millions of tourists in each year, which led to a high consumption of CFI and DEET. The local aquaculture farmers indicated that the waters from the nearby rivers/lakes were utilized as the water supply for the aquaculture ponds. After the water has been used in aquaculture, it was directly discharged into nearby rivers/lakes. Thus, the wastewater from aquaculture could be another source of the PPCP pollution in this area, which should be carefully considered in future work. In the present study, we further divided the sampling sites into agricultural and built-up lands according to their land use types. Generally, the mean concentrations in the agricultural land were lower than those in the built-up land (Figure S2). The result indicates that the urbanization involving more intense human activities would lead to higher loads of PPCPs and this issue is discussed in greater detail in the following text. We noticed that in the autumn, the pollution level of rivers located in the agricultural land (385.9 ± 306.5 ng/L) was significantly higher than that of build-up land (178.1 ± 72.8 ng/L) (*p* < 0.05). This may be caused by some sites with quite high PPCP concentrations (e.g., R1 and R6) in the agricultural land, which was also reflected by the high variations in the sites in the agricultural land. The result suggests that the PPCP pollution in the agricultural land should not be neglected, as a quite high PPCP load could occur in a certain area and period.

Regarding spatial distribution patterns of the PPCPs in Taihu Lake (Figure 3B), the mean concentration of PPCPs in water samples from Zhushan Bay (L1, 96.0 ± 5.9 ng/L) were higher than those from South Coast (L13–L15, 75.4 ± 63.9 ng/L), Gonghu Bay (L4–L5, 56.6 ± 30.8 ng/L), Meiliang Bay (L2–L3, 53.1 ± 37.7 ng/L), Lake Center (L16–L18, 48.0 ± 24.6 ng/L), Wuli Lake (L6, 47.8 ± 16.1 ng/L), and Xishan Island (L19–L20, 28.1 ± 21.5 ng/L). Previous researches have revealed that the PPCP levels were high in the northern part of Taihu Lake [22,52,53], particularly in Zhushan and Meiliang bays, which is consistent with our conclusions. Zhushan Bay is in the northwest part of Taihu Lake, close to Wujing District. It is easily polluted by wastewater and waste from industry and daily life in this very industrialized district. In addition, Zhushan Bay is an semienclosed area, making it difficult to exchange water with the Yangtze River or the center of the lake [22]. Therefore, the PPCPs in Zhushan Bay are hard to be transferred to other areas, thereby leading to high pollution levels. Our study showed relatively high concentrations of PPCPs in southwest Taihu Lake. The distribution of PPCPs in Taihu Lake is strongly linked to natural factors, such as wind wave and hydraulic disturbance. Pollutants may converge or disperse at specific points due to lake flow and topography. Huang et al. [54] concluded that pollutants may converge in the southwest coastal areas of Taihu Lake (L11, L12, and L14) under the action of circulation. Moreover, the PPCP load of the inflow rivers in the southwest coastal areas may be high, although this area is beyond the research area of the present study.

#### 3.3.2. Influence of Urbanization Level on the Distribution of PPCPs in the River Networks

**Figure 4:**
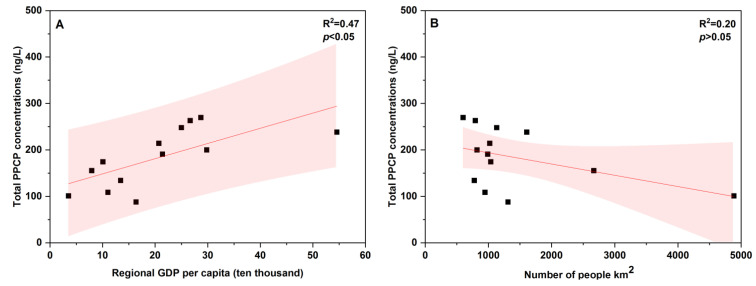
Relationship between concentrations of PPCPs and (**A**) GDP and (**B**) population density in the TLB. Note: the shaded area denotes 95% prediction interval, and the demographic and economic data were obtained from the latest official statistical yearbook; per capita GDP was calculated from resident population.

The generally high level of PPCP pollution in built-up land suggests that urbanization may be an important factor affecting the spatial distribution of PPCPs in the TLB. To further address this issue, we have explored the relationship between per capita GDP and permanent population density and the concentrations of PPCPs in each administrative region in the northern TLB, since economy and population are considered as important indicators of urbanization. The demographic and economic data used in this study were obtained from the official statistical yearbook. As shown in Figure 4A, the PPCP pollution was positively correlated with per capita GDP in the river water of northern TLB (*p* < 0.05). The result was in accordance with the study of Shao et al. [55] for 22 cities in China, indicating that per capita GDP was a feasible indicator to trace PPCP pollution in the northern TLB. Similarly, Santos et al. [56] reported that the higher values of GDP per capita are associated with the higher pharmaceutical pollution in Brazil. This is expected as a higher income and greater awareness of personal health and hygiene of people may contribute to higher PPCP consumption and thus more severe pollution [56]. However, we fail to establish relationship between population density and PPCP pollution (*p* > 0.05) in this study (Figure 4B). As shown in Figure 4B, there is no obvious difference in population density across the regions within the northern TLB, with the population densities in most of the regions around 1000 people km^−2^.

### 3.4. Source Apportionment and Annual Flux Estimation of PPCPs in the Northern TLB

Researchers have suggested that during wastewater treatment, if CBZ shows high persistency and CFI is easily degraded, the CFI/CBZ ratio can be adopted to indicate the proportion of untreated wastewater contributing to the PPCP pollution in nature waters [24,38]. In view of the results from the WWTP removal rates as mentioned above, the CFI/CBZ ratio is suitable to be applied in the present study. Generally, a ratio >10 indicates the low efficiency of sewage treatment and the direct discharge of a large proportion of domestic sewage into receiving waters, and a ratio >50 indicates direct discharge of domestic sewage into the surface water or the existence of unknown sewage sources [38]. In the present study, the ratios of CFI/CBZ of different sites in the river network ranged from 0.3 to 220.7, with a mean value of 21.1. This fraction pattern was apparently different from the WWTP effluents (0.4–2.9) but similar to the influents (6.9–144.4), indicating that direct discharges of domestic wastewater or unidentified source of untreated water are likely the main contributors for the PPCP pollution of the rivers in the northern TLB. In addition, the ratios of CFI/CBZ in different sites of Taihu Lake ranged from 0.1 to 95.1, with a mean value of 4.9, which was lower than that in river waters of northern TLB. This difference suggests that the lake area is more dilute than the river entering it from the streams impacted by untreated wastewater.

The PCA-MLR model was employed to further quantitatively describe the source of PPCPs in the river water of northern TLB, which has been successfully used for source identification of PPCPs in river waters [26,27]. PPCPs without detection or with low detection frequency were excluded from this section. Two principal components (PC1 and PC2) were identified after varimax rotation using PCA, which accounted for 29.0% and 24.8% of the total variance, respectively (Table 4). PC1 had high loading of ROX and CLR, which have low removal rates in our study. Thus, high proportions of these compounds would represent a source from treated water or naturally attenuated untreated water. PC2 is highly associated with GFB, DEET, and BZB. In our study, the removal efficiencies of GFB, DEET, and BZB by WWTPs were generally >60%, suggesting these compounds can be removed in WWTPs. Thus, PC2 can be highly associated with the untreated water source [27]. MLR analysis of elements in the factor scores matrix (*t_k_*) against the normal standard deviate of the SumPPCP values ($\hat{Z}$) was performed on the PCA scores to determine the mass apportionment of the two components in all river samples. According to Equation (1), the resulting equation was:

(4) $$\overset{}{{\overset{\wedge }{Z}}_{\mathrm{SumPPCP}}}=0.253t_{1}+0.727t_{2}\;(R^{2}=0.59,\;p\;<\;0.05)$$

Thus, the mean percentage contribution calculated by Equation (2) were 74.2% for untreated water source (*t*_2_), and 25.8% for treated water source (*t*_1_), respectively. Overall, more than 70% of the PPCP burden in northern TLB was from untreated water.

**Figure 5:**
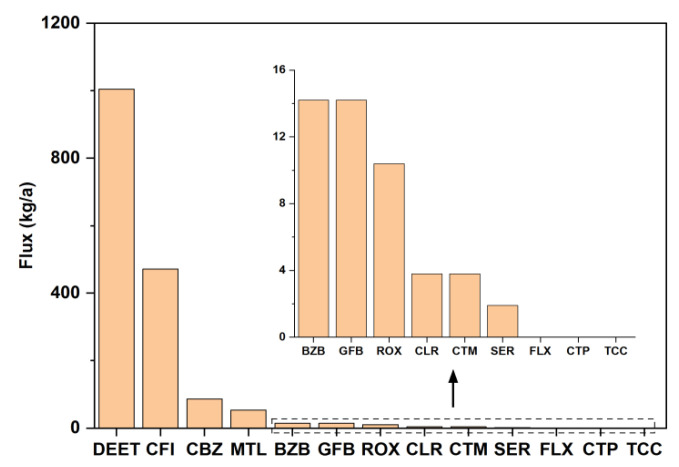
The mean annual fluxes of 13 PPCPs from the inflow rivers in the northern TLB to Taihu Lake in 2021. The water discharge date of the inflow rivers was obtained from the government hydrologic website (http://www.tba.gov.cn/slbthlyglj/sj/sj.html, accessed on 4 July 2022).

In order to understand the actual pollution load of PPCPs in the northern TLB, we estimate the annual flux of PPCPs. The mean annual fluxes of the 13 PPCPs from northern TLB to Taihu Lake are shown in Figure 5. The total annual flux of 13 PPCPs from the inflow rivers to northern Taihu Lake in 2021 was in the range of 0.4–5.3 t/a, with the mean value of 1.6 t/a, which was two orders of magnitude higher than that from the effluent of WWTPs to rivers in this study (16.5 kg/a). Among the detected antibiotics, the mean annual flux of ROX to Taihu Lake (10.4 kg/a) was considerably lower than that from the Yangtze Estuary into coastal waters (0.03–0.66 t/a) [57], and it was similar to that reported in Wujin District (7.2 kg/a) [24]. The mean annual flux of CLR (3.8 kg/a) was observed at a median level in comparison with that of the others. It was 3.8 times higher than that reported in Sweden (1.0 kg/a) [42] and similar to that reported in Wujin District (4.4 kg/a) [24]. For non-antibiotic pharmaceuticals, the mean annual fluxes of MTL, BZB, GFB, CFI, and CBZ were similar to those in the Wujin District [24] and Shijing River [31]. In addition, the mean annual flux of DEET (982.6 kg/a) in this study was considerably higher than that in the Wujin District (76.05 kg/a) [24] and Sweden (0.7 kg/a) [42]. PPCP fluxes depend on both concentration and water flow in the river. For example, the mean concentration of CBZ (12.9 ng/L) corresponds approximately to that of the Ugie River Basin (11.2 ng/L) [30]. However, the mean annual flux of CBZ in this study (73.9 kg/a) was one order of magnitude higher than that in the Ugie River Basin (7.5 kg/a). Therefore, PPCPs concentrations alone cannot represent actual pollution load, and it is necessary to estimate PPCPs fluxes to understand the pollution load of these PPCPs from different sources into an aquatic environment.

## 4. Conclusions

This study performed a comprehensive analysis of the occurrences, source, and flux of typical PPCPs across different water bodies from land waters to the open lake area. The results showed that the concentrations of detected PPCPs were typically in the ng/L level. Some non-therapeutic pharmaceuticals, including CFI and DEET, were the main components in the northern TLB and the lake area. The effluents of WWTPs were not the main contributor for the PPCP pollution in the river network, whereas the untreated wastewater accounted for more than 70% of the PPCP pollution in the river waters. The riverine input governs the seasonal and spatial distributions of PPCP pollution in the Taihu Lake, with an annual input flux of 0.4–5.3 t/a. For local policymakers, it is very urgent to control the PPCP pollution by cutting down the direct discharges of personal care products (e.g., CFI and DEET) from untreated wastewaters to the Taihu Lake through the river networks in the TLB.

## Figures and Tables

**Table 1:** Range and mean concentrations (ng/L) of PPCPs in all the influent and effluent samples of WWTPs.

PPCPs	WWTP A Influent (ng/L)			WWTP A Effluent (ng/L) (OD)			WWTP A Effluent (ng/L) (MBR)			WWTP B Influent (ng/L)			WWTP B Effluent (ng/L) (A^2^O)		
	Range	Mean	Median	Range	Mean	Median	Range	Mean	Median	Range	Mean	Median	Range	Mean	Median
ROX	10.7–12.4	11.6 ± 1.2	11.6	35.1–55.9	45.5 ± 14.7	45.5	ND–32.0	32.0	32.0	ND–7.2	3.9 ± 4.6	3.9	9.3–31.3	20.3 ± 15.6	20.3
CLR	ND	ND	ND	9.3–16.3	12.8 ± 4.9	12.8	0.2–9.3	4.8 ± 6.4	4.8	0.1–0.8	0.5 ± 0.5	0.9	2.7–5.5	4.1 ± 2.0	4.1
FLX	ND	ND	ND	ND	ND	ND	ND	ND	ND	ND	ND	ND	ND	ND	ND
CTP	ND	ND	ND	2.1–4.9	3.5 ± 2.0	3.5	ND–3.0	3.0	3.0	0.5–0.8	0.7 ± 0.2	ND	ND–0.1	0.1 ± 0.1	0.1
SER	ND	ND	ND	ND–0.1	0.1	0.1	ND-–0.3	0.3	.03	ND	ND	ND	ND	ND	ND
MTL	101.4–152.3	126.9 ± 36.0	126.9	187.6–224.4	206 ± 26.0	206.0	32.4–80.7	56.6 ± 34.2	56.6	8.4–10.4	9.4 ± 1.4	9.4	ND–4.6	2.3 ± 3.3	2.3
BZB	3.2–4.3	3.7 ± 0.8	3.8	ND	ND	ND	ND	ND	ND	0.7–2.1	1.4 ± 0.9	0.7	ND–1.7	0.9 ± 1.2	0.9
GFB	ND	ND	ND	ND	ND	ND	ND	ND	ND	3–4.4	3.7 ± 1.0	3.7	ND–1.3	0.7 ± 0.9	0.7
DEET	70.8–703.3	387.1 ± 447.2	387.1	11.5–23.5	17.5 ± 8.4	17.5	20.80–44.4	32.6 ± 16.9	32.6	3.0–72.6	37.8 ± 49.2	44.3	14.0–272.2	143.1 ± 182.6	54.6
TCC	ND	ND	ND	ND	ND	ND	ND	ND	ND	ND	ND	ND	ND	ND	ND
CTM	ND	ND	ND	ND	ND	ND	ND	ND	ND	ND	ND	ND	ND–0.1	0.1 ± 0.1	0.1
CFI	630.4–1608.8	1119.6 ± 691.8	1119.6	12.7–14.7	13.7 ± 1.4	13.7	6.9–28.10	17.5 ± 15.0	17.5	86.9–97.9	92.4 ± 7.8	92.4	15.9–25.9	21.0 ± 7.7	21
CBZ	7.7–13.3	10.5 ± 4.0	10.5	16.4–26.1	21.3 ± 6.9	21.3	6.5–17.6	12.1 ± 7.8	12.1	ND–11.9	6.0 ± 8.4	5.9	8.9–41.0	25.0 ± 22.7	25.0

ND: not detected, less than the limit of detection.

**Table 2:** Detection frequency and concentration range of PPCPs in the river water.

PPCPs	Range (ng/L)	Mean (ng/L)	Median (ng/L)	Freq (%)
ROX	ND–28.3	2.6 ± 4.4	0.7	95.6
CLR	ND–8.1	1.0 ± 1.3	0.1	70.6
FLX	ND	ND	ND	0.0
CTP	ND–2.0	1.4 ± 0.3	ND	4.0
SER	ND–0.7	0.3 ± 0.1	ND	12.4
MTL	ND–26.5	4.5 ± 4.6	2.8	94.6
BZB	ND–13.8	2.3 ± 2.3	0.6	69.1
GFB	ND–2.9	1.0 ± 0.6	ND	29.1
DEET	5.5–971.2	92.0 ± 145.7	55.7	100.0
TCC	ND	ND	ND	0.0
CTM	ND–1.4	0.4 ± 0.3	ND	52.4
CFI	8.5–807.8	76.0 ± 123.5	41.4	100.0
CBZ	ND–116.7	12.0 ± 21.8	4.1	97.1

ND: not detected, less than the limit of detection.

**Table 3:** Detection frequency and concentration range of the PPCPs in Taihu Lake.

PPCPs	Range (ng/L)	Mean (ng/L)	Median (ng/L)	Freq (%)
ROX	ND–13.3	1.9 ± 3.3	0.4	79.5
CLR	ND–0.4	0.2 ± 0.1	0.2	29.5
FLX	ND	ND	ND	ND
CTP	ND–1	0.4 ± 0.3	0.3	18.0
SER	ND–113.3	3.3 ± 17.2	0.4	56.4
MTL	ND–8.5	1.6 ± 1.9	0.9	65.4
BZB	ND	ND	ND	0.0
GFB	ND	ND	ND	0.0
DEET	0.2–80.8	15.9 ± 12.3	15.3	100.0
TCC	ND	ND	0.0	0.0
CTM	ND–0.9	0.3 ± 0.3	0.2	6.4
CFI	ND–169.4	22.7 ± 29.5	11.9	98.7
CBZ	ND–24.3	7.6 ± 4.2	7.1	89.7

ND: not detected, less than the limit of detection.

**Table 4:** Varimax-rotated component matrix following principal component analysis of all river water samples. Bold values denote the absolute value of PCA loading higher than 0.6.

Variable	Rotated Component Number	
	1	2
ROX	**0.959**	0.007
CLR	**0.978**	0.011
MTL	0.010	0.361
BZB	0.445	**0.807**
GFB	0.509	**−0.698**
DEET	−0.075	**0.883**
CTM	0.074	0.127
CFI	0.392	−0.206
CBZ	−0.009	0.040
Variance explained	29.0%	24.8%

## Supplementary Materials

The following supporting information can be downloaded at: https://www.mdpi.com/article/10.3390/ijerph191711135/s1, Figure S1: Schematic diagrams of the treatment processes in the two WWTPs; Figure S2: PPCP concentrations in the different land use types in four seasons; Table S1: Physicochemical property and molecular of target PPCPs; Table S2: Each sampling site with corresponding river and sub-region of Taihu Lake; Table S3: Parameter settings of solid phase extraction; Table S4: Mobile phase elution procedures; Table S5: Mass spectrometry conditions for 13 PPCPs; Table S6: The mean removal rates of PPCPs in three processes used by two WWTPs.
