# Supplementary material for: Pharmaceuticals and Personal Care Products across Different Water Bodies in Taihu Lake Basin, China: Occurrence, Source, and Flux
Source: Int J Environ Res Public Health. 2022 Sep 5;19(17):11135. doi: 10.3390/ijerph191711135 (PMC9517866; doi:10.3390/ijerph191711135)
Supplement: Supplementary file 1 [file ijerph-19-11135-s001.zip › ijerph-1884296-supplementary.pdf]

Pharmaceuticals and Personal Care Products across Different Water Bodies in Taihu Lake Basin, China: Occurrence, Source, and Flux

Supplemental Material:

Content:

SM-1	Figure S1. Schematic diagrams of the treatment processes in the two WWTPs.
SM-2	Figure S2. PPCP concentrations in the different land use types in four seasons.
SM-3	Table S1. Physicochemical property and molecular of target PPCPs.
SM-4	Table S2. Each sampling site with corresponding river and sub-region of Taihu Lake.
SM-5	Table S3. Parameter settings of solid phase extraction.
SM-6	Table S4. Mobile phase elution procedures.
SM-7	Table S5. Mass spectrometry conditions for 13 PPCPs.
SM-8	Table S6. The mean removal rates of PPCPs in three processes used by two WWTPs.

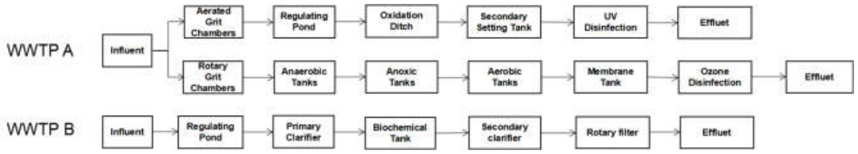

Figure S1. Schematic diagrams of the treatment processes in the two WWTPs.

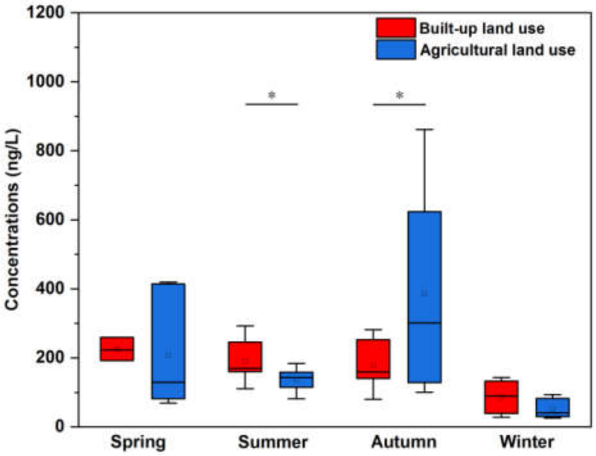

Figure S2. PPCP concentrations in different land use types in four seasons. The symbols "*" indicate statistically significant difference at 0.05 level (two-tailed).

Table S1. Physicochemical property and molecular of target PPCPs.

Compound	Abbreviation	CAS Number	Molecular Formula	Relative Molecular Mass	logK_{ow}
roxithromycin	ROX	80214-83-1	$C_{41}H_{76}N_2O_{15}$	837.05	3.1
clarithromycin	CLR	81103-11-9	$C_{38}H_{69}NO_{13}$	747.95	3.2
fluoxetine	FLX	54910-89-3	$C_{17}H_{18}F_3NO$	309.33	4.0
citalopram	CTP	219861-08-2	$C_{20}H_{21}FN_2O$	324.39	3.2
sertraline	SER	79617-96-2	$C_{17}H_{17}Cl_2N$	306.23	4.8
metoprolol	MTL	37350-58-6	$C_{15}H_{25}NO_3$	267.36	1.9
bezafibrate	BZB	41859-67-0	$C_{19}H_{20}ClNO_4$	361.82	3.8

gemfibrozil	GFB	25812-30-0	$C_{15}H_{22}O_3$	250.33	3.8
n, n-diethyl-m-toluamide	DEET	134-62-3	$C_{12}H_{17}NO$	191.27	2.0
triclocarban	TCC	101-20-2	$C_{13}H_9Cl_3N_2O$	315.58	5.3
clotrimazole	CTM	23593-75-1	$C_{22}H_{17}ClN_2$	344.84	5.0
caffeine	CFI	58-08-2	$C_8H_{10}N_4O_2$	194.19	-0.1
carbamazepine	CBZ	298-46-4	$C_{15}H_{12}N_2O$	236.27	2.5

Table S2. Each sampling site with corresponding river and sub-region of Taihu Lake.

Environmental Compartments	Sampling Sites	Location
WWTPs	WWTP A	120.3343ºE,31.6107ºN
	WWTP B	120.8467ºE,31.6123ºN
Rivers	R1	120.8296ºE,31.7805ºN
	R2	120.7985ºE,31.7611ºN
	R3	120.6433ºE,31.6518ºN
	R4	120.5998ºE,31.5379ºN
	R5	120.5709ºE,31.5044ºN
	R6	120.4193ºE,31.4515ºN
	R7	120.8985ºE,31.7806ºN
	R8	120.7622ºE,31.5992ºN
	R9	120.5057ºE,31.3343ºN
	R10	120.8223ºE,31.6938ºN
	R11	120.3573ºE,31.4734ºN
	R12	120.1278ºE,31.5071ºN
	R13	120.0371ºE,31.4989ºN
	R14	120.0102ºE,31.4604ºN

	R15	120.0106ºE,31.4281ºN
	R16	119.9536ºE,31.3603ºN
	R17	119.9315ºE,31.3212ºN
Taihu Lake	L1	120.0472ºE,31.4258ºN
	L2	120.1728ºE,31.4686ºN
	L3	120.1622ºE,31.3966ºN
	L4	120.2462ºE,31.3789ºN
	L5	120.3757ºE,31.4478ºN
	L6	120.2453ºE,31.5156ºN
	L7	120.2292ºE 31.3103ºN
	L8	120.2697ºE 31.2328ºN
	L9	120.4590ºE 31.1717ºN
	L10	119.9639ºE 31.3111ºN
	L11	119.9583ºE 31.2167ºN
	L12	120.0119ºE 31.1364ºN
	L13	120.1347ºE 30.9931ºN
	L14	120.0257ºE 31.0664ºN
	L15	120.2996ºE 31.0017ºN
	L16	120.0969ºE 31.3333ºN
	L17	120.1033ºE 31.2258ºN
	L18	120.1505ºE 31.0628ºN
	L19	120.2676ºE 31.0136ºN
	L20	120.1897ºE 31.1347ºN

Table S3. Parameter settings of solid phase extraction.

**Operation**	**Solvent**	**Flow（mL/min）**	**volume（mL）**
Condition	methanol	5	5
	ultrapure water	5	5
Sample loading	sample	10	1000
Elute	methanol	5	5
	Methanol:acetone (v/v 1:1)	5	5

Table S4. Mobile phase elution procedures.

**Gradient（min）**	**Flow（mL/min）**	**Mobile Phase A %**	**Mobile Phase B %**
0	0.4	90	10
0.25	0.4	90	10
3.00	0.4	5	95
4.00	0.4	5	95
4.01	0.4	90	10
5	0.4	90	10

Mobile phase A: 0.1% formic acid; Mobile phase B: 100% acetonitrile.

Table S5. Mass spectrometry conditions for 13 PPCPs.

Compound	Retention Time（ms）	Precursor Ion (m/z)	Product Ions (m/z)	Cone Voltage (V)	Collision Energy (eV）	Modes
ROX	7	837.50	158.0	37	30	ESI+
CLR	7	748.40	158.20	40	30	ESI+
FLX	7	310.13	43.99	6	10	ESI+
			147.99		8	
CTP	8	325.10	108.93	38	26	ESI+
			262.03		20	
SER	11	306.10	158.86	20	26	ESI+
			274.97		12	
MTL	36	268.2	116.0	30	18	ESI+
			133.0		24	
BZB	27	360.27	153.90	26	26	ESI-
			274.03		16	
GFB	70	251.03	82.95	18	12	ESI+
			128.93		10	
DEET	27	192.13	90.93	38	28	ESI+
			118.95		18	
TCC	70	314.97	161.85	28	16	ESI-
			162.17		26	
CTM	17	277.07	160.94	42	20	ESI+
			164.98		22	
CFI	45	195.07	110.03	40	24	ESI+
			138.04		22	
CBZ	7	236.97	165.0	30	42	ESI+
			178.87		34	

Table S6. The mean removal rates of PPCPs in three processes used by two WWTPs.

Compound	Removal Rates (%) in WWTP A		Removal Rates (%) in WWTP B
	OD Process	MBR Process	A^2O Process
ROX	–289.4	–29.1	–892.4
CLR	/	/	–1593.8
MTL	–66.2	57.6	77.9
BZB	100.0	100.0	–142.9
GFB	/	/	56.7
DEET	82.6	82.2	–320.8
CFI	98.6	97.6	77.6
CBZ	–104.6	–38.8	–244.5
